# Responses of soil microbial community diversity to thinning and replanting, and their effects on microbial necromass carbon accumulation efficiency

**DOI:** 10.3389/fmicb.2026.1875448

**Published:** 2026-06-26

**Authors:** Haitong Huang, Yaxuan Sun, Xinyue Chen, Huiling Zhou, Sining Liu, Hongwei Xu

**Affiliations:** Forest Ecology and Conservation in the Upper Reaches of the Yangtze River Key Laboratory of Sichuan Province, Sichuan Mt. Emei Forest Ecosystem National Observation and Research Station, College of Forestry, Sichuan Agricultural University, Chengdu, China

**Keywords:** broadleaf, coniferious, forest management, microbial activity, soil carbon cycling, thinning

## Abstract

Thinning and replanting of broadleaf trees (TRBT) significantly affect stand structure and soil functions, but their impacts on soil microbial community diversity and enzyme activity remain poorly understood. This study evaluated five different TRBT treatments: unthinning plot (NT), thinning without replanting (NP), thinning with replanting of *Schima superba* (CS), thinning with replanting of *Liquidambar formosana* (CL), and thinning with replanting of both *Schima superba* and *Liquidambar formosana* (CSL). We analyzed soil microbial community structure, diversity, and enzyme activity, and explored their relationships with microbial necromass carbon (MNC) accumulation efficiency. TRBT mainly affected bacterial diversity, while having minimal impacts on fungal diversity and richness. TRBT significantly altered the β-diversity of both bacterial and fungal communities. The *Acidobacteriia, Alphaproteobacteria, Deltaproteobacteria, Ktedonobacteria*, and *Gammaproteobacteria* were the dominant bacterial classes, while the *Agaricomycetes* were the dominant fungal classes. TRBT reduced the coefficients of β-1,4-glucosidase (BG), cellobiohydrolase (CBH) and acid phosphatase (AP), but increased the coefficients of β-1,4-N-acetylglucosaminidase (NAG) and leucine aminopeptidase (LAP). Meanwhile, the soil bacterial diversity and coefficients of NAG and LAP in CS and CL treatments were higher than those in CSL, whereas the coefficients of BG and CBH in CS treatment were lower than those in Cl and CSL. The MNC accumulation coefficient was significantly positively correlated with bacterial diversity and NAG coefficient, but negatively correlated with coefficients of BG, CBH, and AP. Mantel test revealed that microbial biomass carbon (MBC), phosphorus (MBP), enzyme activity coefficients (CBH, AP, NAG), bacterial diversity, and *Alphaproteobacteria* (bacterial) were key factors influencing the MNC accumulation coefficient. Random forest analysis further confirmed that MBP, coefficients of AP and NAG, and MBN were important variables for predicting the MNC accumulation coefficient. Our findings provide a valuable reference for optimizing tree species selection to improve soil carbon sequestration in plantation forests.

## Introduction

*Cunninghamia lanceolata* is a fast-growing, high-quality afforestation species with both economic and ecological value (Wu et al., 2021). However, current *Cunninghamia lanceolata* plantations are pure forest (Guo et al., 2022), which commonly face ecological challenges such as poor stand quality, soil degradation, declining biodiversity, and imbalanced nutrient cycling (Ding et al., 2023). To address these issues, the practice of thinning and replanting of broadleaf trees (TRBT) has been widely implemented to improve stand structure and enhance forest quality (Huang et al., 2025; Zhao et al., 2025a).

Soil microorganisms play a crucial role in the biogeochemical cycling within forest ecosystems (Baldrian, 2017). Numerous studies have confirmed that soil microbes decompose plant and animal residues and transform soil organic matter, actively participating in the cycling of carbon, nitrogen, phosphorus, and other nutrients, thus forming the foundation for maintaining functional stability in forest ecosystems (Schimel and Schaeffer, 2012). Meanwhile, the diversity and structural complexity of soil microbial communities are increasingly recognized as key determinants of ecosystem multifunctionality, with more complex microbial networks conferring greater functional stability and resilience (Zhang et al., 2025; Wang et al., 2026). Shifts in community composition and the enrichment of key functional groups accelerate organic matter decomposition and nitrogen-carbon cycling, thereby promoting the restoration of degraded forest ecosystems (Zhang et al., 2025).

Soil enzyme activity, closely linked to microbial function, serves as a key biological indicator reflecting the intensity and direction of soil ecological processes (Blonska et al., 2020). As metabolic products of microbial activity, soil enzymes directly influence nutrient transformation efficiency and the rate of organic matter decomposition (Trivedi et al., 2016). Given the central role of soil microorganisms and enzyme activity in forest ecosystems, a thorough understanding of their dynamic changes and interactive relationships under TRBT is essential for revealing the mechanisms underlying ecosystem function maintenance and assessing soil carbon cycling. However, traditional absolute enzyme activities may be affected by concurrent changes in microbial biomass under thinning and replanting, potentially obscuring shifts in microbial metabolic investment. The soil enzyme activity coefficient (SEAC), defined as enzyme activity per unit of microbial biomass carbon or nitrogen, overcomes this limitation by directly reflecting the enzyme productivity or nutrient acquisition efficiency per unit biomass, thereby eliminating the confounding effect of simple biomass variation. By capturing the relative microbial demand for carbon and nitrogen, SEAC can more accurately reveal alterations in microbial community metabolic strategies and nutrient requirements (Qu and Hai, 2025). Critically, SEAC has been demonstrated to significantly correlate with microbial necromass carbon accumulation, explaining a substantial portion of its variance (Qu and Hai, 2025). Therefore, integrating SEAC with microbial community diversity under TRBT may offer a more precise understanding of how silvicultural practices regulate microbial-driven carbon sequestration efficiency.

At present, a large number of studies have investigated the effects of TRBT on soil microbial diversity and enzyme activity, with most studies indicating a positive impact (Huang et al., 2022; Li et al., 2022). The underlying mechanism is generally attributed to improved stand structure, which optimizes light availability, moisture conditions, and nutrient cycling—thereby creating a more favorable environment for soil microbes (He et al., 2025; Yu et al., 2025)—and subsequently promotes microbial growth and increased enzyme activity (Wang et al., 2023). However, conflicting evidence exists. Some studies report that the removal of above-ground biomass during thinning significantly reduces litter input to the forest floor (Zhao et al., 2025b). Since litter serves as a primary source of food and energy for soil microbes, its reduction can directly constrain microbial metabolic activity (Fanin et al., 2021; Zheng et al., 2021), leading to suppressed microbial diversity, particularly among fungal communities (Zheng et al., 2024). Furthermore, microenvironmental changes induced by TRBT may disrupt the equilibrium of the original microbial community, causing sensitive taxa to decline or perish, thereby exacerbating negative impacts (Wang et al., 2026). Additionally, some research has found no significant difference in microbial properties or enzyme activities between mixed and pure stands (Manral et al., 2020), likely due to the inherent stability and self-regulatory capacity of soil microbial systems (Zhao et al., 2025a). Collectively, these findings highlight substantial uncertainty regarding the overall effects of TRBT on soil microbial diversity and enzyme activity.

Soil microorganisms, through growth, reproduction, and eventually death, contribute to the accumulation of soil organic carbon (SOC) as necromass has become a key focus in current soil carbon cycling research (Liang et al., 2019; Zhu et al., 2020). The evidence confirms that microbial necromass carbon (MNC) is one of the most stable components of the SOC pool, contributing over 50% to total SOC in many ecosystems (Wang et al., 2021). It plays a critical role in maintaining soil carbon sink capacity and ecosystem stability (Liang et al., 2017). To quantify the MNC accumulation efficiency, researchers have proposed the MNC accumulation coefficient—defined as the amount of MNC produced per unit of microbial biomass (Wang et al., 2021). This metric directly reflects the capacity of microbial communities to convert their biomass into stable organic carbon. A higher value indicates greater MNC accumulation per unit of microbial biomass, implying stronger carbon retention efficiency by the microbial community (Wang et al., 2021). While numerous studies have established that soil microbial diversity and enzyme activity are key factors driving changes in MNC (Zhou et al., 2025; Xu et al., 2026), how these factors influence the MNC accumulation coefficient remains unclear.

Therefore, in this study, five treatments [unthinning plot (NT), thinning without replanting (NP), thinning with replanting of *Schima superba* (CS), thinning with replanting of *Liquidambar formosana* (CL), and thinning with replanting of both *Schima superba* and *Liquidambar formosana* (CSL)] were selected to investigate the effects of TRBT on soil microbial community diversity and enzyme activity coefficients, and explore their relationships with MNC accumulation efficiency. We hypothesized that: (1) TRBT significantly increases soil bacterial and fungal diversity, elevates soil enzyme activity coefficients, and modulates the composition and structure of soil microbial communities; (2) CSL exhibits higher soil microbial diversity and enzyme activity; and (3) soil bacterial and fungal diversity and soil enzyme activity coefficients are key factors driving changes in MNC accumulation efficiency.

## Methods

### Study sites

The study site is located in Hongya Forest Farm, Meishan City, Sichuan Province (29°43′-29°48′ N, 103°09′-103°14′ E), with an average elevation of 1200–1300 m. The mean annual temperature and precipitation are 16.6 °C and 1435.5 mm, respectively, and the soil type is mountainous yellow soil. The *Cunninghamia lanceolata* plantation in the study area is approximately 16–20 years old and was initially a monoculture. To improve stand quality, selective thinning of dominant trees was conducted from September to December 2020. In January to February 2021, native broadleaf tree species, *Schima superba* and *Liquidambar formosana*, were planted as understory seedlings.

### Experimental design and sample collection

This study implemented four thinning and replanting of broadleaf trees (TRBT) treatments: thinning without replanting (NP), thinning with replanting of *Schima superba* (CS), thinning with replanting of *Liquidambar formosana* (CL), and thinning with replanting of both *Schima superba* and *Liquidambar formosana* (CSL). An unthinning plot (NT, control) was established as the control. Each treatment had three replicate plots, and within each plot, three 20 × 20 m subplots were set up, resulting in a total of 45 subplots. In August 2023, soil samples were collected from the 0–10 cm layer using a soil auger following an “S” pattern. The soil sample was divided into three portions: one air-dried for analysis of basic physicochemical properties, one stored at 4 °C for determination of soil microbial biomass and enzyme activities, and one kept at −80 °C for microbial community structure and diversity analysis.

### Laboratory analyses

Soil organic carbon (SOC), total nitrogen (TN), and total phosphorus (TP) were determined using the Walkley and Black, Kjeldahl, and molybdenum blue methods, respectively (Bremner and Mulvaney, 1982; Nelson and Sommers, 1982). Soil microbial biomass carbon (MBC), nitrogen (MBN), and phosphorus (MBP) were determined by the chloroform fumigation-extraction method (Vance et al., 1987). Extracellular enzyme activities were measured using a 96-well microplate fluorometric assay (Saiya-Cork et al., 2002), including β-1,4-glucosidase (BG), cellobiohydrolase (CBH), β-1,4-N-acetylglucosaminidase (NAG), leucine aminopeptidase (LAP), and phosphatase (AP). The analysis of soil microbial community structure and diversity was conducted with support from Rhonin Biosciences (http://rhonin-bio.com/). Among them, the U515F (5′-GTGCCAGCMGCCGCGG-3′) and U806R (5′-GGACTACHVGGGTWTCTAAT-3′) primer pairs with barcodes were used to amplify the bacterial specific V4 hyper variable region of the 16S rRNA gene, ITS3 (5′-GATGAAGAACGYAGYRAA-3′) and ITS4 (5′-TCCTCCGCTTATTGATATGC-3′) primers were applied to ITS2 region amplicon. Detailed methods for analyzing soil enzyme activity, microbial biomass, microbial necromass carbon (MNC), Phospholipid fatty acid (PLFA), and microbial community were referenced from Zhou et al. (2025).

## Statistical analysis

The soil enzyme activity coefficient, a metric reflecting both microbial enzyme synthesis efficiency and their corresponding nutrient demand profiles, was calculated as Equation 1


$$\begin{array}{l} \text{Soil enzyme activity coefficient}=\\ \frac{\text{Carbon}/\text{Nitrogen}/\text{Phosphorus enzyme activity }}{\text{MBC}/\text{MBN}/\text{MBP}} \end{array}$$ (1)

MNC coefficient was used to characterize the accumulation efficiency of microbial necromass, which was calculated as Equations 2–5 (Wang et al., 2021):


$$\begin{array}{l} \text{MNC accumulation coefficient}=\frac{\text{MNC}}{\text{PLFA}} \end{array}$$ (2)

$$\begin{array}{l} BNC=MurA\times 45 \end{array}$$ (3)

$$\begin{array}{l} FNC=\left(\frac{GluN}{179.17}-\frac{MurA}{251.23}\times 2\right)\times 179.17\times 9 \end{array}$$ (4)

$$\begin{array}{l} MNC=BNC+FNC \end{array}$$ (5)

where MurA and GluN represent the contents of muramic acid and glucosamine, respectively; 179.17 and 251.23 are their molar masses; 2 is the bacterial GluN/MurA molar ratio (2:1); 45 and 9 are conversion factors for MurA-to-bacterial NC and GluN-to-fungal NC, respectively.

The response ratio (RR) was calculated as Equation 6


$$\begin{array}{l} \text{RR}=\frac{M_{t}\;-\;M_{ck}}{M_{ck}}*100\% \end{array}$$ (6)

where *M*_t_ and *M*_ck_ represent the index in TRBT treatment and control, respectively.

One-way ANOVA was used to assess differences in bacterial and fungal diversity and richness, relative abundance (at the class level), enzyme activity coefficients, and MNC accumulation coefficients among different treatments, with Duncan's *post-hoc* test applied for significance analysis (*p* < 0.05). Only the bacterial taxa with a relative abundance greater than 0.03 were included in the analysis. For fungi, only Agaricomycetes was analyzed, as it exhibited high abundance and significant differences among treatments, whereas other fungal groups showed low abundance and no significant variation across treatments. Non-metric multidimensional scaling (NMDS) was used to study the effects of TRBT on microbial community β-diversity (based on Bray–Curtis dissimilarity). Linear regression analysis was used to evaluate the relationship between the MNC accumulation coefficient and microbial diversity, as well as soil enzyme activity coefficients. Mantel test and random forest analysis were employed to investigate the effects of basic soil physicochemical properties and microbial activity indicators on the MNC accumulation coefficient.

## Results

## Soil bacterial and fungal diversity

**Figure 1:**
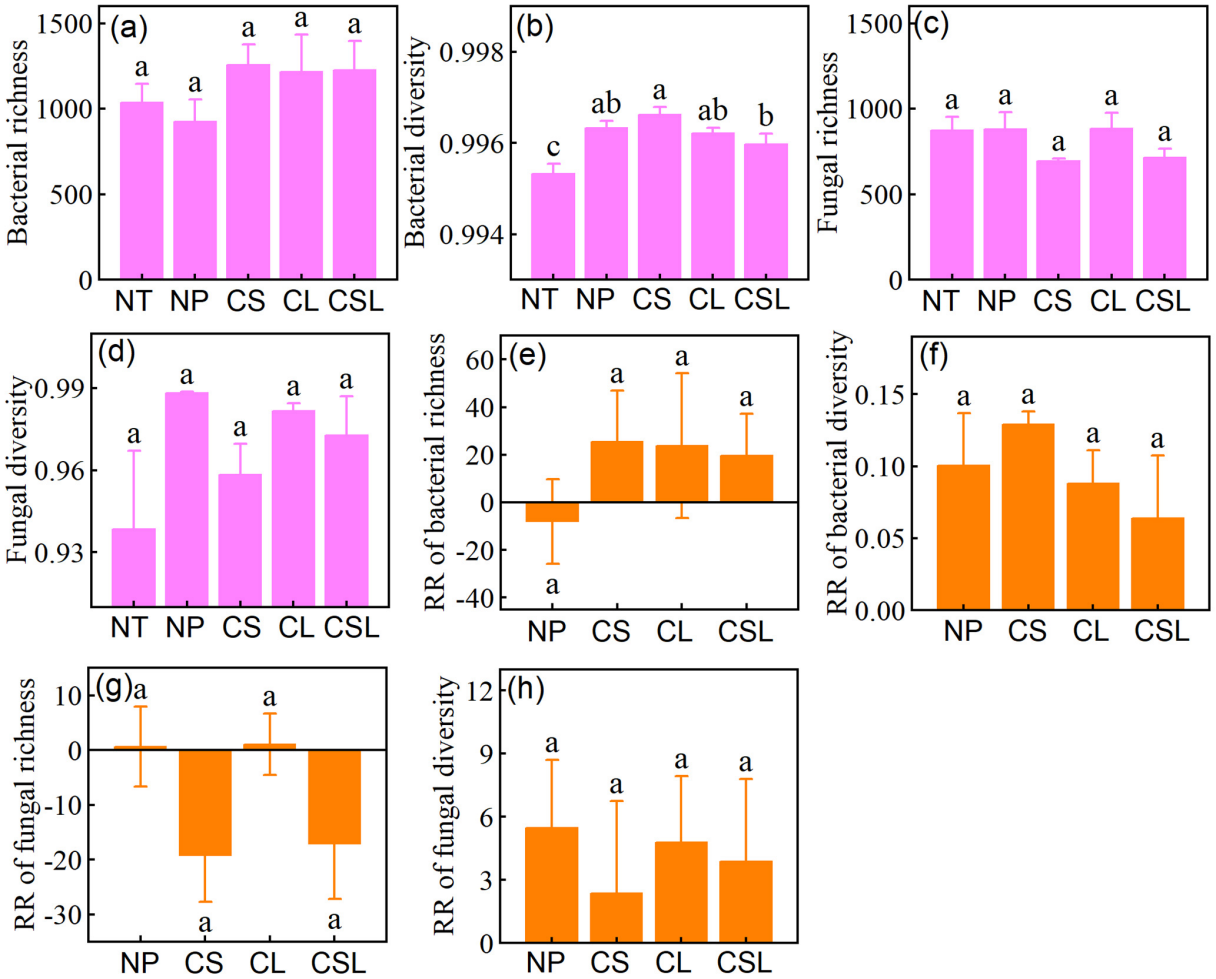
Effects of thinning and replanting on bacterial richness and diversity **(a, b)**, fungal richness and diversity **(c, d)**, and response ratio (RR, %) of bacterial richness and diversity **(e, f)**, fungal richness and diversity **(g, h)**. NT, unthinning plot (control); NP, thinning without replanting; CS, thinning with replanting of *Schima superba*; CL, thinning with replanting of *Liquidambar formosana*; CSL[[Inline Image]], thinning with replanting of both *Schima superba* and *Liquidambar formosana*. Lowercase letters indicate significant differences between different treatments at *p* < 0.05.

The thinning and replanting of broadleaf trees (TRBT) had no significant effect on bacterial and fungal richness, and fungal diversity, but the bacterial diversity in thinning without replanting (NP), thinning with replanting of *Schima superba* (CS), thinning with replanting of *Liquidambar formosana* (CL), and thinning with replanting of both *Schima superba* and *Liquidambar formosana* (CSL) treatments was significantly higher than that in unthinning plot (NT) (Figures 1a–d). There were no significant differences in RR of richness and diversity of bacteria and fungi in different treatments, and the RR of bacterial and fungal diversity were all positive (Figures 1e–h).

## Soil microbial abundance and structure

**Figure 2:**
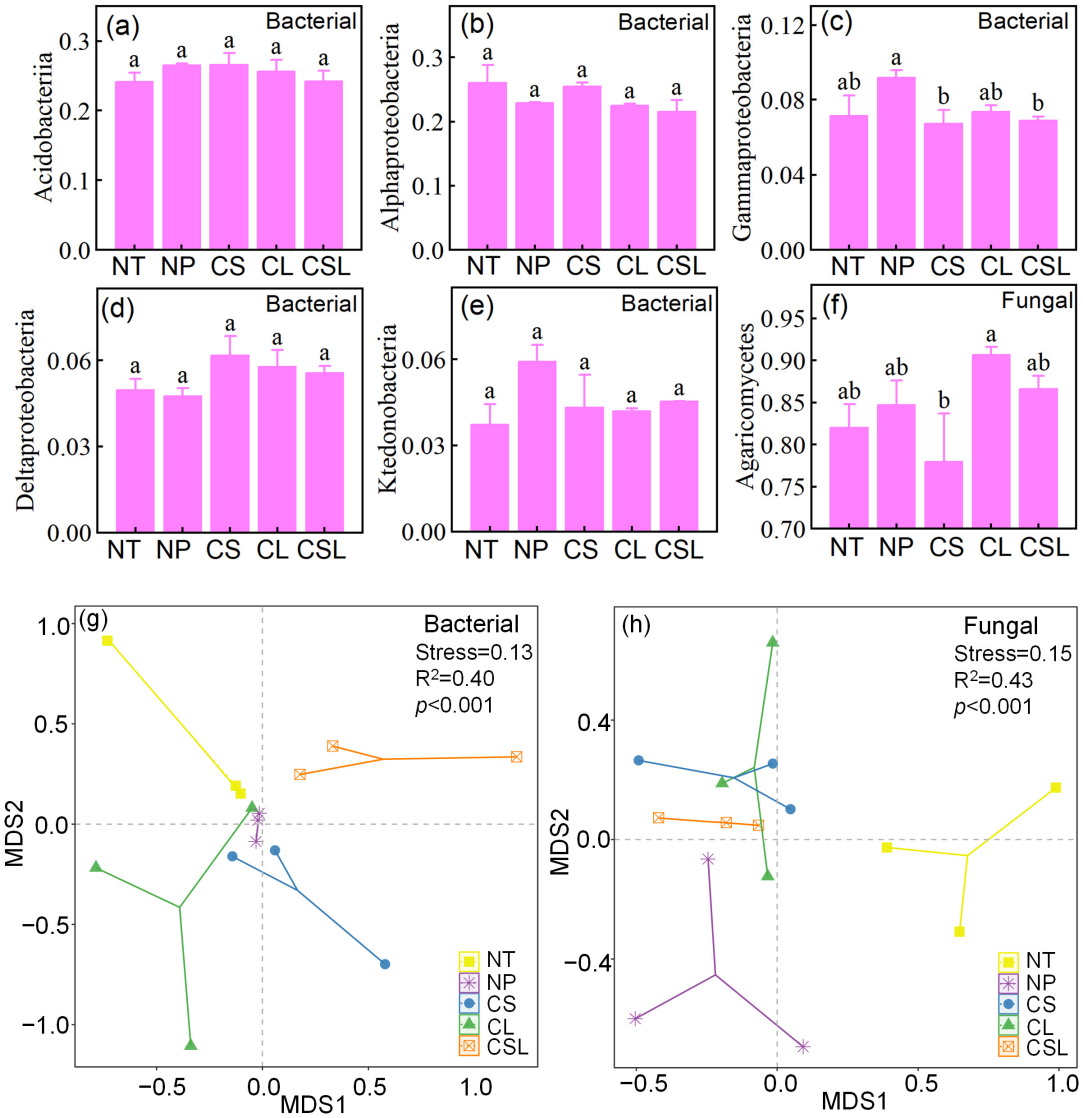
Mean (± SE) of relative abundance (class level) of soil bacterial **(a–e)** and fungal **(f)**, and structure of soil bacterial **(g)** and fungal **(h)**. Treatment codes are shown in Figure 1.

Five bacterial classes (*Acidobacteriia, Alphaproteobacteria, Deltaproteobacteria, Ktedonobacteria*, and *Gammaproteobacteria*) dominated across all treatments (Figures 2a–e). TRBT did not significantly alter the relative abundance of the first four. However, *Gammaproteobacteria* were less abundant in the CS and CSL treatments compared to NT. *Agaricomycetes* was the dominant fungal class across all treatments (Figure 2f), and its relative abundance was higher in CL than in NT.

The non-metric multidimensional scaling (NMDS) showed that the dispersion degree of bacterial and fungal communities among different treatments was relatively high. The TRBT treatment was significantly separated from the control (NT), indicating that TRBT significantly affected the community structure of soil bacteria and fungi. However, NP, CS, and CL did not show significant separation in the bacterial community, and CS, CL, and CSL did not show significant separation in the fungal community, suggesting that there were no significant differences in the bacterial and fungal community structures of these treatments (Figures 2g, h).

## Soil enzyme activity coefficient and microbial necromass carbon (MNC) accumulation coefficient

**Figure 3:**
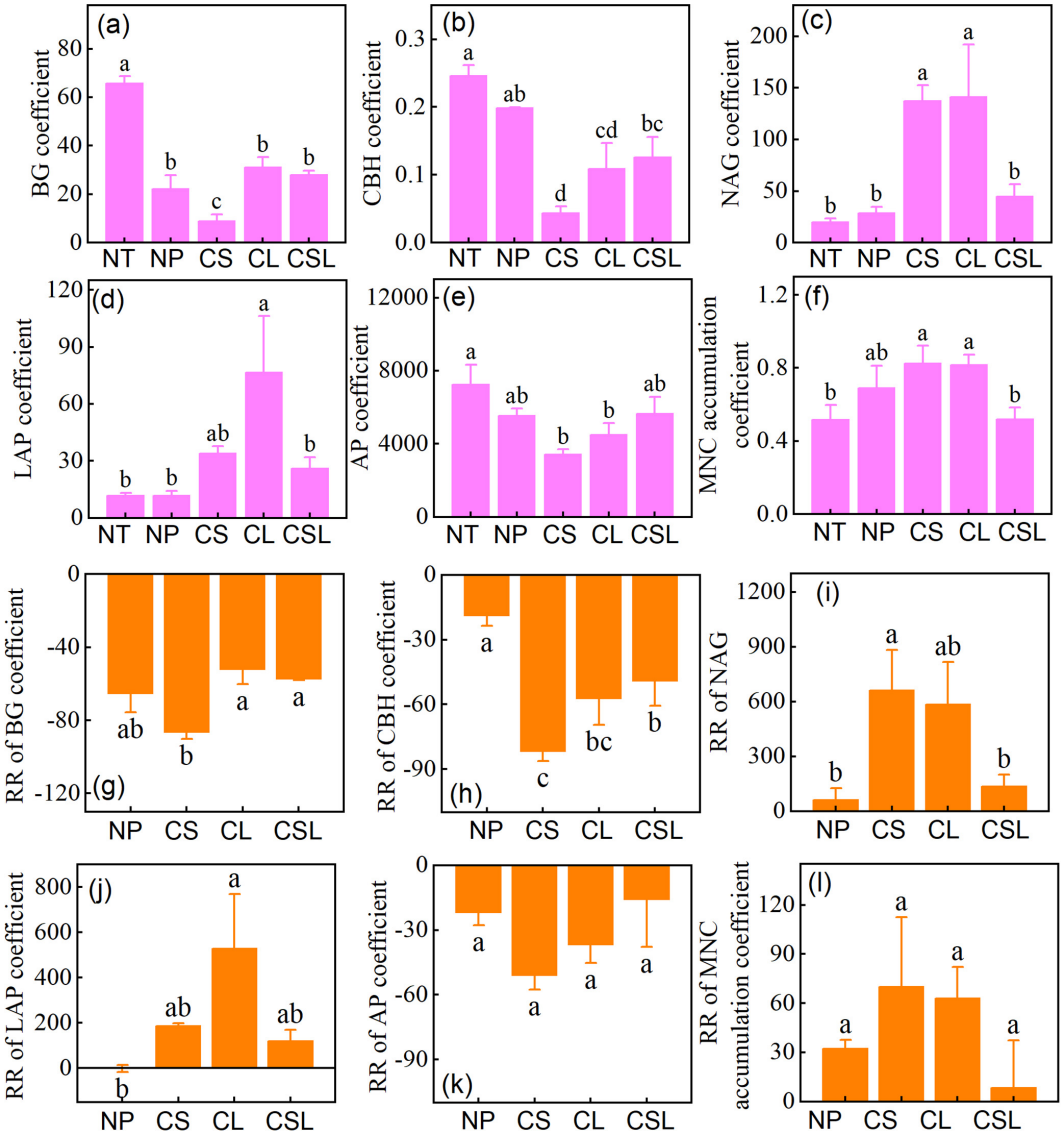
Mean **(a–f)** and response ratio (RR, %; **g–l**) of soil enzyme activity coefficient and microbial necromass carbon (MNC) accumulation coefficient. Treatment codes are shown in Figure 1. BG, β-1, 4-glucosidase. CBH, β-D-cellobiosidase. NAG, β-1, 4-N-acetylglucosaminidase. LAP, L-leucine aminopeptidase. AP, acid phosphatase.

The β-1,4-glucosidase (BG), cellobiohydrolase (CBH) and acid phosphatase (AP) coefficient in NP, CS, CL, and CSL treatments were lower than those in NT, but the β-1,4-N-acetylglucosaminidase (NAG) and leucine aminopeptidase (LAP) coefficient were higher in CS and CL treatments than those in NT (Figures 3a–e). Meanwhile, the MNC accumulation coefficient was higher in NP, CS, and CL treatments than that in NT (Figure 3f). In adition, the RR of BG, CBH, and AP coefficient of all treatments were all negative, but the NAG coefficient and MNC accumulation coefficient were positive (Figures 3g–l).

## Relationship of MNC accumulation coefficient with bacteria and fungal diversity

**Figure 4:**
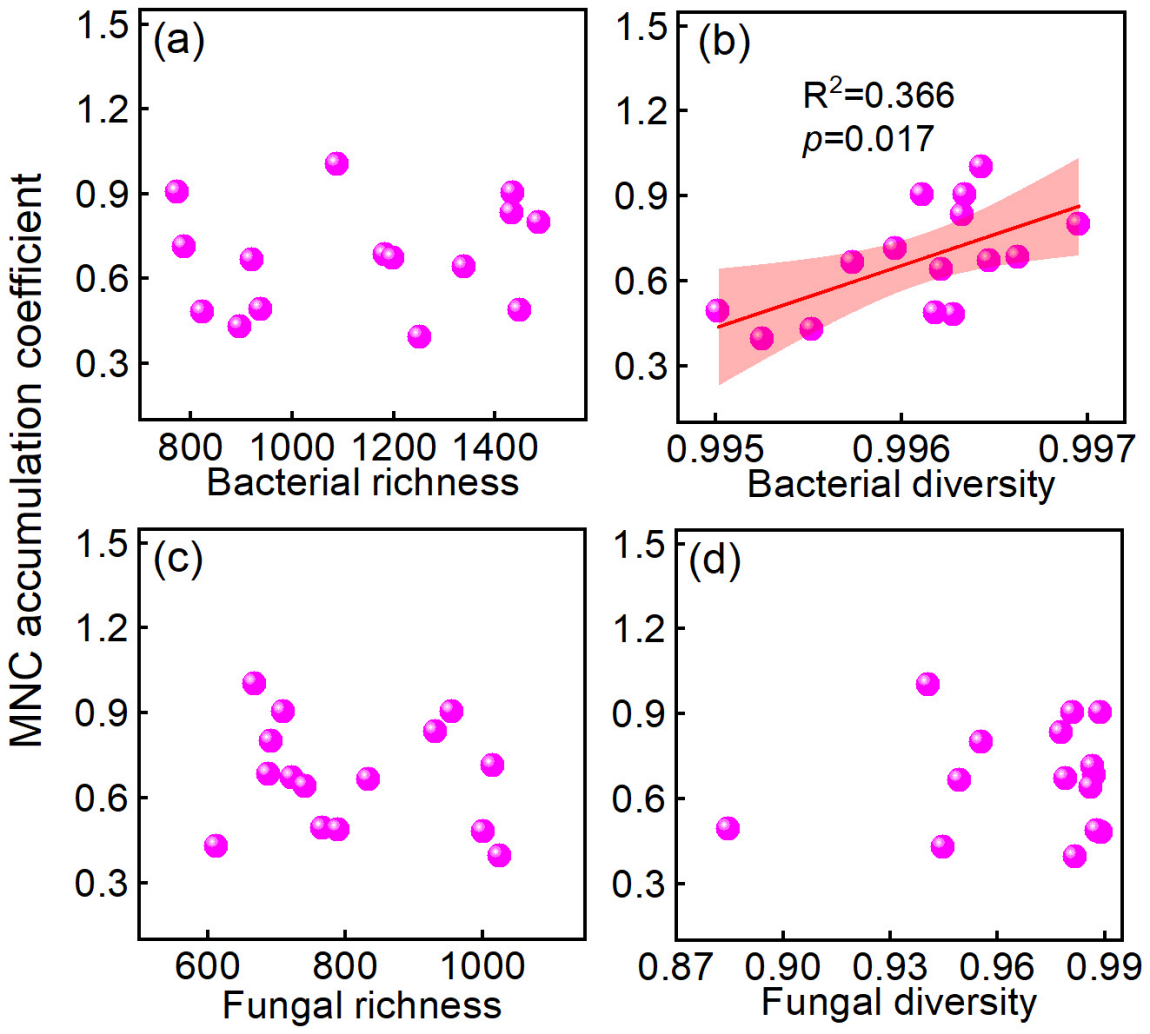
Relationship of microbial necromass carbon (MNC) accumulation coefficient with bacterial **(a, b)** and fungal **(c, d)** diversity.

**Figure 5:**
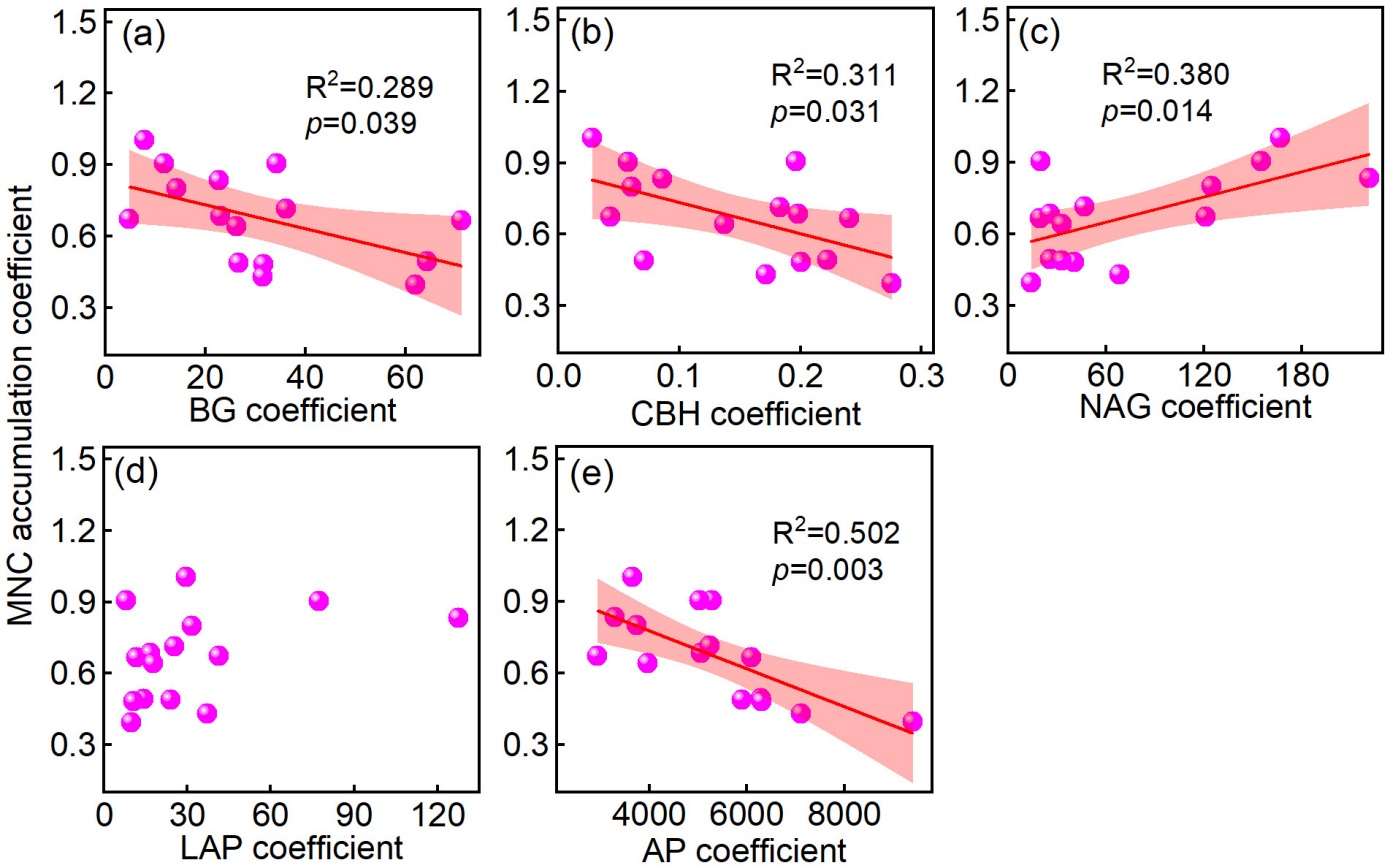
Relationship of microbial necromass carbon (MNC) accumulation coefficient with soil enzyme activity coefficient of BG **(a)**, CBH **(b)**, NAG **(c)**, LAP **(d)**, and AP **(e)**. Treatment codes are shown in Figure 3.

A significant positive correlation was observed between the MNC accumulation coefficient and bacterial diversity, whereas no significant correlations were detected with bacterial richness, fungal richness, or fungal diversity (Figures 4a–d). Furthermore, the MNC accumulation coefficient exhibited significant negative correlations with the BG, CBH, and AP coefficients, a significant positive correlation with the NAG coefficient, and no significant correlation with the LAP coefficient (Figures 5a–e).

**Figure 6:**
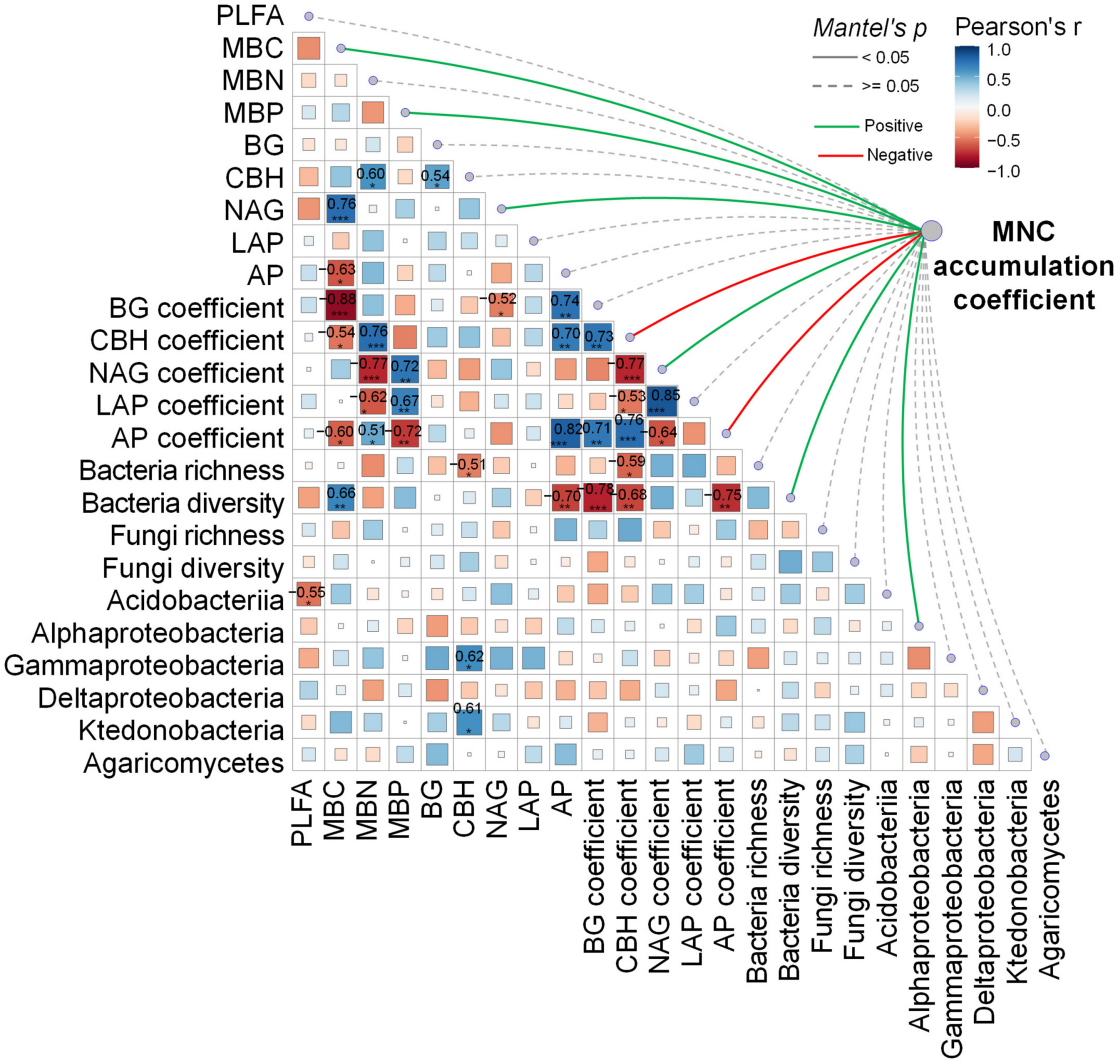
Main predictor importance (% of increase of MSE) of soil microbial biomass, enzyme activity, enzyme activity coefficient, diversity and abundance of bacterial and fungal on microbial necromass carbon (MNC) accumulation coefficient by random forest modeling analysis. ***p < 0.001; **p < 0.01; *p < 0.05.

Mantel test analysis revealed that MBC, MBP, NAG, CBH coefficient, NAG coefficient, AP coefficient, bacterial diversity, and *Alphaproteobacteria* (bacterial) were key factors influencing the MNC accumulation coefficient. Specifically, the MNC accumulation coefficient showed significant positive correlations with MBC, MBP, NAG, NAG coefficient, bacterial diversity, and *Alphaproteobacteria* (bacterial), while it was significantly negatively correlated with the CBH coefficient and AP coefficient (Figure 6).

**Figure 7:**
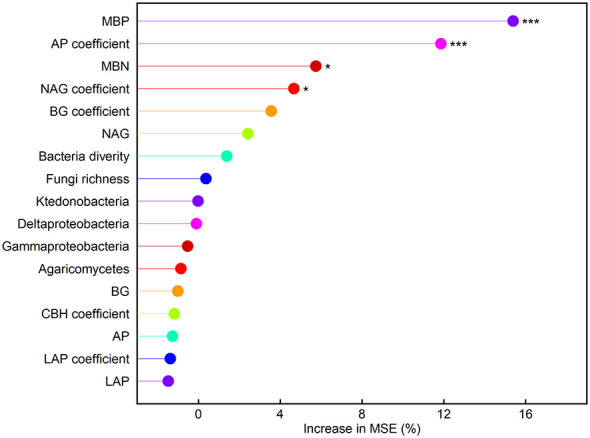
Effects of soil microbial biomass, enzyme activity, enzyme activity coefficient, and bacterial and fungal diversity and abundance on microbial necromass carbon (MNC) accumulation coefficient based on the Mantel test. Treatment codes are shown in Figure 3. PLFA, phospholipid fatty acid. MBC, microbial biomass carbon. MBP, microbial biomass phosphorus. ***p < 0.001; *p < 0.05.

Random forest analysis further confirmed that MBP, AP coefficient, MBN, and NAG coefficient are important variables for predicting the MNC accumulation coefficient, with MBP and AP coefficient being the top predictive factors (Figure 7).

## Discussion

### Effect of thinning and replanting of broadleaf trees (TRBT) on soil microbial diversity

Our study showed that TRBT significantly increased bacterial diversity. Although there were no significant differences in bacterial richness and fungal diversity indices between the thinning with replanting of *Schima superba* (CS), thinning with replanting of *Liquidambar formosana* (CL), and thinning with replanting of both *Schima superba* and *Liquidambar formosana* (CSL), and unthinning plot (NT) treatments, the response ratios for both bacterial richness (19.59%−25.46%) and fungal diversity (2.38%−4.80%) were positive, indicating a trend toward enhancement. Meanwhile, non-metric multidimensional scaling (NMDS) analysis reveals that TRBT significantly influenced bacterial and fungal β-diversity. This study indicated that TRBT is a key driver of bacterial diversity and community structure. Our findings further support the established understanding that TRBT can markedly enhance soil microbial activity and reshape functional characteristics (Li et al., 2021; Tan et al., 2025).

Soil microbial diversity and community composition are primarily regulated by the integrated effects of plant litter input, soil physical structure, and microhabitat conditions (Buyer et al., 2010; Qiqige et al., 2025). First, TRBT optimizes stand density, thereby improving the composition and stability of soil aggregates (He et al., 2025). Soil aggregates not only provide a physical refuge for microorganisms, but also offer favorable living conditions due to their rich nutrient content (Hou et al., 2021; da Costa Soares et al., 2026). Concurrently, improved soil aeration and water retention following the TRBT enhance the thermal and moisture conditions favorable for microbial growth, indirectly boosting community diversity and metabolic activity (Wang et al., 2026). Moreover, as a key management strategy in plantation cultivation, TRBT significantly improved understory light availability and microclimatic conditions by reducing canopy closure (Zhao et al., 2025a). Increased light intensity can directly affect microbial metabolic processes and, more importantly, indirectly drive shifts in microbial community structure by altering the composition and quantity of root exudates from plants (Zhang et al., 2025).

This study highlights that bacteria exhibit significantly higher sensitivity to TRBT than fungi, likely due to fundamental differences in their ecological strategies and physiological traits. Bacteria are predominantly *r*-strategists. They are characterized by rapid reproduction and strong adaptability. These traits enable swift responses to environmental changes (Kushwaha et al., 2018). In contrast, fungi are typically *k*-strategists, with slower growth rates and greater community stability, resulting in weaker short-term responses to disturbances (Cycon et al., 2017). Additionally, differences in resource utilization strategies may further explain their divergent responses to TRBT (Wang et al., 2026). This rapid bacterial response to labile carbon input from broadleaf litter may partially explain the greater variation we observed in bacterial community structure and the shifts in enzyme activity coefficients following TRBT. After forest thinning, the type and quality of litter input change, and the introduction of broadleaved species adds new carbon sources—such as labile broadleaf litter (Zhao et al., 2025a). Bacteria, being more efficient at utilizing simple organic compounds (e.g., sugars and amino acids), can rapidly proliferate when resource availability increases (Gul et al., 2024). Fungi, although capable of decomposing complex substrates like lignin, may show less pronounced short-term changes, leading to relatively stable community dynamics.

### Effect of TRBT on soil microbial composition

The *Acidobacteriia, Alphaproteobacteria, Deltaproteobacteria, Ktedonobacteria*, and *Gammaproteobacteria* were the dominant bacterial classes of all treatments. *Acidobacteriia* are widely distributed in acidic soils (Islam et al., 2022). Although litter input changed after TRBT, the long-accumulated acidic organic matter in *Cunninghamia lanceolata* plantation has not been fully mineralized (Zhao et al., 2025a), which favors the proliferation of *Acidobacteriia*. Meanwhile, broadleaf litter is rich in labile sugars, phenolic compounds, polysaccharides, and nitrogen-containing compounds (Terajima and Moriizumi, 2013), and releases a large amount of soluble carbon during the initial decomposition phase (Terajima and Moriizumi, 2013), providing more energy and substrate resources for *Alphaproteobacteria, Ktedonobacteria*, and *Gammaproteobacteria*. In addition, while TRBT improves overall soil aeration, localized anaerobic microzones persist beneath the litter layer, offering favorable habitats for *Deltaproteobacteria*. The *Agaricomycetes* was the dominant fungal class. This group plays a crucial role in forest soil nutrient cycling and represents the largest and most ecologically diverse class within the phylum *Basidiomycota* (Qiao et al., 2025). Its ecological dominance is attributed to strong environmental adaptability and exceptional capacity to degrade lignocellulose (Pent et al., 2017). With the replanting of broadleaf trees, root exudates—such as sugars and organic acids—provide additional carbon sources (Terajima and Moriizumi, 2013), further stimulating the colonization and expansion of *Agaricomycetes*.

### Effect of TRBT on soil enzyme activity coefficient

Overall, TRBT reduced the activity coefficients of carbon-acquiring enzymes (BG and CBH) and phosphorus-acquiring enzyme (AP). This pattern is primarily attributed to the fact that the TRBT induced a greater increase in microbial biomass carbon (MBC) and phosphorus (MBP) than in the production of carbon- and phosphorus-acquiring enzymes (Figure S1; Figure S2). The introduction of broadleaved species significantly altered the diversity and quality of litter inputs (Huang et al., 2025). Coniferous litter was typically coarse, lignin-rich, and high in tannins, resulting in slow decomposition rates. In contrast, broadleaf litter was rich in labile carbohydrates, providing higher-quality substrates for microbial utilization (Terajima and Moriizumi, 2013). When “food and energy” resources are abundant, microbes reduce their investment in producing extracellular enzymes for breaking down recalcitrant carbon (e.g., lignin, cellulose), leading to lower carbon-acquisition enzyme activity. Moreover, broadleaf litter generally has a lower carbon-to-phosphorus (C:P) ratio than coniferous litter, indicating relatively higher phosphorus content per unit of litter (Pang et al., 2020). This helps alleviate soil phosphorus limitation, thereby reducing microbial demand for phosphorus-acquisition enzymes and their secretion efficiency. In contrast, TRBT increased nitrogen-acquiring enzyme activity coefficients (NAG and LAP), indicating enhanced microbial investment in nitrogen acquisition. These findings suggest that during the TRBT process, microbial demand for carbon and phosphorus decreased, while demand for nitrogen increased.

### Effect of broadleaf tree types on soil microbial diversity and enzyme activity coefficient

The broadleaf tree types is a critical factor governing soil microbial diversity and enzyme activity (Qu et al., 2025; Zhao et al., 2025a). This study compared different conifer-broadleaf mixed planting configurations and found that the soil bacterial diversity and the coefficients of nitrogen-acquiring enzymes (NAG and LAP) in treatments with single-species replanting of *Schima superba* or *Liquidambar formosana* were generally higher than in the mixed-replanting treatment. The *Schima superba* is an arbuscular mycorrhizal (AM) tree species, while *Liquidambar formosana* is ectomycorrhizal (EcM). When co-planted, these species may release allelopathic compounds—such as phenolics and terpenoids—that mutually inhibit root-associated microbes (Qin et al., 2018), potentially disrupting rhizosphere microbial community structure. Meanwhile, mixed planting intensifies competition among roots for water, nutrients, and spatial resources (Xu et al., 2024), which may constrain microbial metabolic investment and reduce extracellular enzyme production efficiency.

### Effect of soil microbial diversity and enzyme activity coefficients on microbial necromass carbon (MNC) accumulation efficiency

Soil microbial biomass (MBC, MBN, and MBP) is a key factor and predictor influencing MNC accumulation efficiency. As microorganisms are the direct source of MNC, their biomass determines the production of MNC (Zhou et al., 2025; Xu et al., 2026). Although many studies have highlighted microbial diversity as an important factor affecting the MNC accumulation (Jiang et al., 2025; Chen et al., 2026), this study found no significant correlation between MNC accumulation efficiency and fungal richness, fungal diversity, or bacterial richness—potentially due to the stable variation of these indices within the study. In contrast, bacterial diversity showed a significant positive correlation with MNC accumulation efficiency, and Mantel tests identified it as one of the primary influencing factors. Increased bacterial diversity implies greater functional group diversity, particularly the enrichment of r-strategist Gram-positive bacteria and cellulose-degrading bacteria (Kushwaha et al., 2018). These taxa are metabolically active and capable of efficiently decomposing low C:N organic matter in broadleaf litter (Cycon et al., 2017), thereby promoting MNC accumulation.

MNC accumulation efficiency decreases with carbon- and phosphorus-acquiring enzyme activity coefficients, and Mantel test analysis reveals that CBH coefficient and AP coefficient are key factors influencing MNC accumulation efficiency, with AP coefficient serving as a significant predictor. This indicates that higher carbon- and phosphorus-acquiring enzyme activities promote the mineralization and decomposition of MNC (Wang et al., 2021). In contrast, the MNC accumulation coefficient increases with nitrogen-acquiring enzyme activity, and the NAG coefficient is a significant driver and predictor, suggesting that enhanced nitrogen-acquiring enzyme promotes MNC accumulation. This is because MNC is a nitrogen-rich substrate, and its synthesis requires greater nitrogen availability (Liang et al., 2020). Notably, the dependence of MNC on phosphorus-acquiring enzyme activity is significantly stronger than on carbon- or nitrogen-acquiring enzymes, likely due to more intense nitrogen limitation in our study region (Zhao et al., 2025a).

### Limitations

Thinning and replanting significantly alters stand structure, canopy light transmission, and other microenvironmental conditions in forest ecosystems (Ding et al., 2023; Huang et al., 2025). Soil microbial communities and enzyme activities are primarily regulated by multiple abiotic factors including soil aggregate structure, moisture content, and temperature (Li et al., 2021; Huang et al., 2022). However, the present study did not systematically evaluate how soil temperature and physical properties mediate microbial activity, which represents a key limitation of our current work. Furthermore, the year after thinning is a critical modulator of soil enzyme dynamics and soil carbon cycling (Qu et al., 2025), as the effects of forest management on below-ground processes typically accumulate over long timescales (Xu et al., 2024). Since the thinning and replanting treatment in this study was implemented relatively recently, long-term continuous monitoring will be required in future research to reveal the general patterns and underlying mechanisms of how this management practice affects soil microbial community assembly and functional traits.

## Conclusion

The sensitivity of bacteria to thinning and replanting of broadleaf trees (TRBT) was significantly higher than that of fungi, but TRBT significantly altered the community structure of both bacteria and fungi. During the TRBT process, microbial demand for carbon and phosphorus decreased, while nitrogen demand increased. Meanwhile, soil bacterial diversity and the coefficients of carbon and phosphorus-related enzymes in treatments with single-species replanting of Schima superba or Liquidambar formosana were generally higher than in the mixed-replanting treatment, whereas the coefficients of nitrogen-related enzymes in the single-species replanting treatments were generally lower than in the mixed-replanting treatment. Furthermore, bacterial diversity and enzyme activity coefficients were key factors influencing MNC accumulation efficiency.

## Data Availability

The original contributions presented in the study are included in the article/Supplementary material, further inquiries can be directed to the corresponding authors.
