# Supplementary material for: Responses of soil microbial community diversity to thinning and replanting, and their effects on microbial necromass carbon accumulation efficiency
Source: Front Microbiol. 2026 Jun 26;17:1875448. doi: 10.3389/fmicb.2026.1875448 (PMC13350042; doi:10.3389/fmicb.2026.1875448)
Supplement: Supplementary file 1 [file Table_1.DOCX]

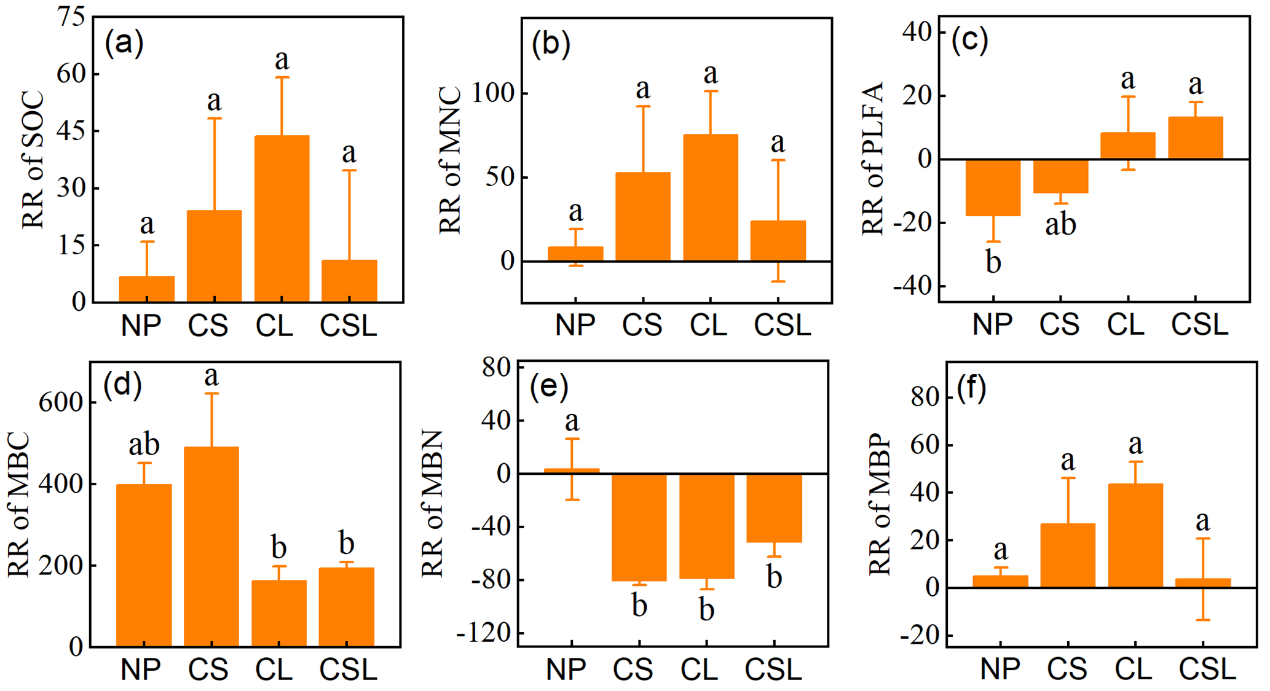


Figure S1 Response ratio (RR, %) of soil organic carbon (SOC), microbial necromass carbon (MNC), phospholipid fatty acid (PLFA), microbial biomass carbon (MBC), microbial biomass nitrogen (MBN), and microbial biomass phosphorus (MBP). Notes: NT, unthinning plot (control); NP, thinning without replanting; CS, thinning with replanting of *Schima superba*; CL, thinning with replanting of *Liquidambar formosana*; CSL, thinning with replanting of both *Schima superba* and *Liquidambar formosana*. Lowercase letters indicate significant differences between different treatments at *p* < 0.05.

**
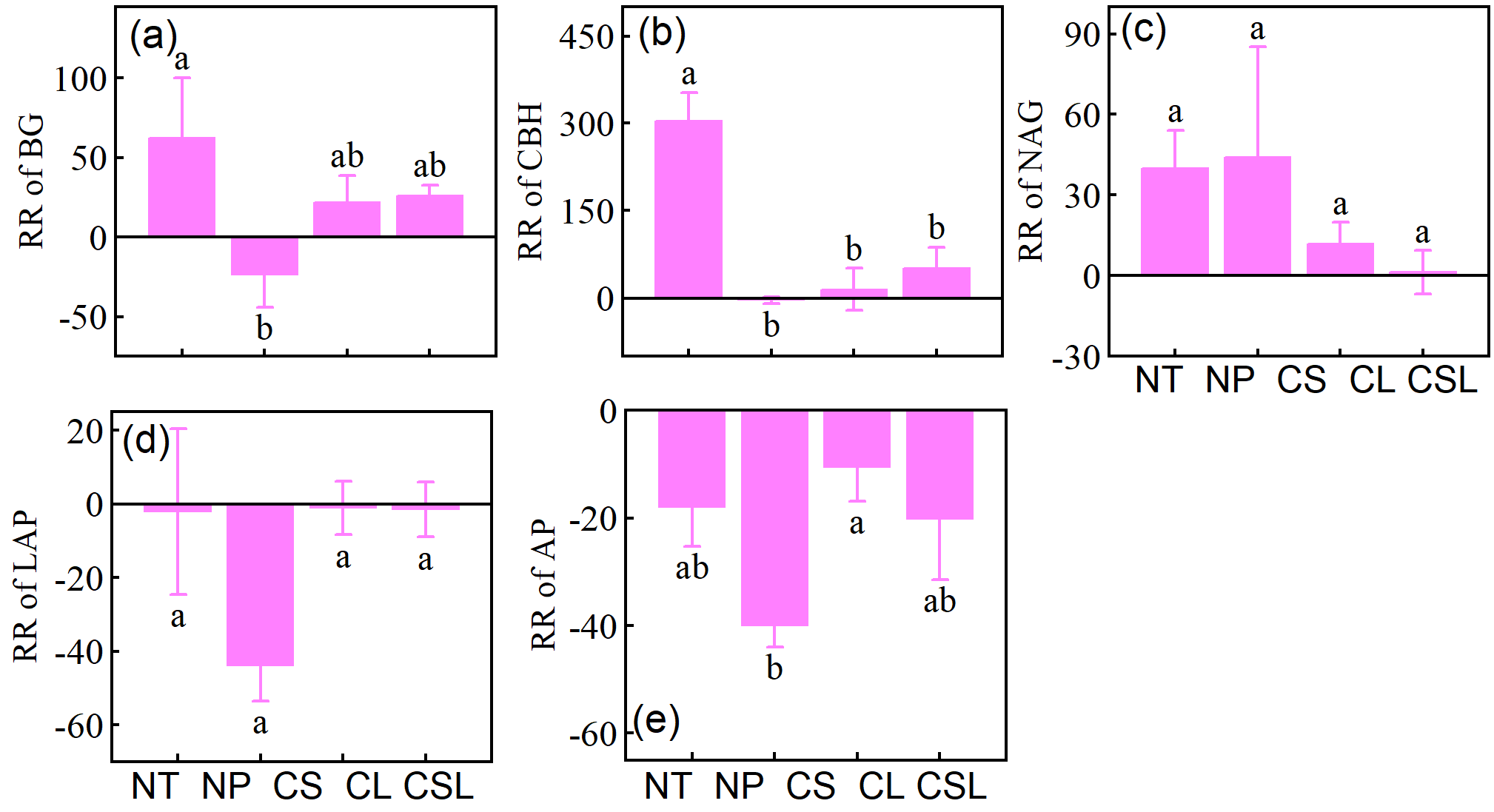
**

Figure S2 Response ratio (RR, %) of soil enzyme activity coefficient. Notes: Treatment codes are shown in FIGURE S1. BG, β-1, 4-glucosidase. CBH, β-D-cellobiosidase. NAG, β-1, 4-N-acetylglucosaminidase. LAP, L-leucine aminopeptidase. AP, acid phosphatase.
